# Prevalence of alcohol consumption and alcohol use disorders among outdoor drinkers in public open places in Nigeria

**DOI:** 10.1186/s12889-018-5344-6

**Published:** 2018-03-27

**Authors:** Victor O. Lasebikan, Olatunde Ayinde, Mayokun Odunleye, Babajide Adeyefa, Samson Adepoju, Shina Fakunle

**Affiliations:** 1 Department of Psychiatry, College of Medicine, University of Ibadan, Ibadan, PMB 5116 Nigeria; 2 Department of Psychiatry, University College Hospital, Ibadan, Nigeria

**Keywords:** Open-place drinking, Harmful use, Hazardous use, Dependence, Alcohol use disorder

## Abstract

**Background:**

There is a rapid shift in the social context of drinking, with a large proportion of regular drinkers favouring outdoor-open space drinking, such as motor-parks, by the road sides, the majority of which are unlicensed premises for drinking.

**Method:**

This study determined the prevalence and determinants of harmful or hazardous alcohol use and possible dependence, defined as a “likely alcohol use disorder” (AUD) in a community sample of 1119 patrons of open space drinking places in Ibadan, Nigeria, using the AUDIT. Scores of 8 and above signified a likely AUD. The associations between a likely AUD and demographic characteristics were sought using Chi square statistics and binary regression analysis was used to determine the effects of multiple confounding variables on a likely AUD using the SPSS version 20.0 software.

**Results:**

Of the entire population, the prevalence of likely AUD was 39.5%, and 44.4% out of the drinking population Multivariate analysis showed that Islamic religion was a negative predictor for likely AUD, OR = 0.13, 95% CI (0.06–0.26), while rural residence, OR = 1.84, 95% CI (1.34–2.53) and cigarette smoking OR = 1.81, 95% CI (1.37–2.40) were predictive of likely AUD.

**Conclusion:**

Outdoor-open space drinkers are likely to have AUD compared with the general population. Open space drinking has a huge public health implication because of the associated health risks and injuries.

## Background

Research evidence from community studies on alcohol indicates that within the last few decades, there has been a rapid increase in alcohol availability [1], production, importation and consumption across all age groups in Nigeria [1–3]. Alcohol consumption is also widely considered as part of social activities in Nigeria and most consumers seldom drink alone [4].

Noticeably, there has been a rapid shift in the social context of drinking with a large proportion of regular drinkers favouring outdoor-open space drinking [5]. Such open-place drinking is at motor-parks, by the road sides, the majority of which are unlicensed premises for drinking [5]. To entice consumers, alcohol vendors provide sources of entertainment such as big televisions for their customers to watch soccer and movies as well as music [1]. These attract drinkers because drinkers choose to drink in the most rewarding contexts, a process that forms the basis for positive self-reinforcement [6].

Factors mediating this social drift from bars and roofed premises to open places are largely unknown. One potential reason may be lack of laws prohibiting open display, sale and consumption of alcohol in open places [7]. The rate of upsurge of these out-door drinking places may indicate that drinking is embedded in Nigerian subcultures such as the Yoruba people of the South-west [3] and the Ijaw people, the Igbo people and the Ibibio people of the South-south of Nigeria [8], and like in some developed countries of the world, may be part of everyday life [4]. Another factor could be the characteristic group-binging of alcohol consumption in Nigeria, which exemplifies one of the activities that might be more permissive in open and outdoor social places [4]. There are indications that the context where drinking occurs may contribute to specific alcohol-related problems [9], and one mechanism by which drinking contexts may contribute to alcohol use and alcohol related-problems is by self-selection of drinkers of a similar drinking pattern.

In Nigeria, the majority of data from epidemiological surveys on alcohol consumption had been drawn from households surveys [3, 10], treatment facilities [11], street children [12, 13] prison population [13], commercial drivers [7] adolescents [14], and the elderly [2]. There is a serious dearth of information on prevalence of drinking in open-places, which patrons constitute a special population at risk of harmful and hazardous drinking, and from whom data obtained may translate into effective policies on problem drinking for them.

Location-based sampling as was done in this research work was believed to be appropriate for obtaining information from people within the ecological contexts of open-drinking to specific levels of health risks. We conceived that such places would be the potential catchment sites of harmful or hazardous drinking pattern in Nigeria who otherwise to the best of our knowledge had never been studied in previous research. Therefore, we determined the prevalence and determinants of harmful or hazardous alcohol use and possible dependence otherwise described as likely Alcohol Use Disorder (AUD) among those that patronized open places for drinking. In this study, we defined open spaces as roofless joints such as motor-parks, by the roadsides or street corners.

## Methods

### Setting of study and background information on the area

The study took place in July 2015 in Ibadan, Nigeria. The city is located in the southwestern part of the country. It has a population of about 2.5 million people as of 2009. Ibadan is divided into eleven local government areas [15].

This report is part of a study that adopted a mixed quantitative and qualitative methodology. The current paper is the report of the quantitative assessment of the study sample.

### Sampling technique and procedure

**Fig. 1:**
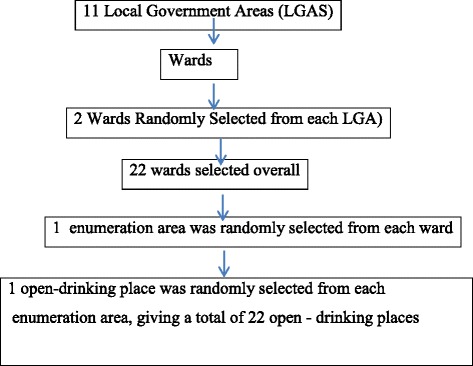
Sample selection flow chart

In this descriptive study, using as a systematic sampling method, 1119 participants were selected. The process of selection is as follows: all the 11 local governments in Ibadan were divided into wards (a ward is a local authority area, typically used for electoral purposes) [16], then, 2 wards were randomly selected in each of the local governments. The wards were then divided into enumeration areas (the smallest geographical unit for an enumerator to cover in order to administer a questionnaire) [16], after which 1 enumeration area was randomly selected from each of the wards and an open drinking place was randomly selected from each enumeration area, thereby giving a total of 22 open drinking places spanning the 11 local governments in the state, the sampling process is illustrated in Fig. 1. We obtained a list of licensed business premises with the state ministry of commerce and with the respective local government. Although the intent of the issued licenses was for indoors sale of alcohol, vendors had generally shifted their business to open places, which their licenses do not cover but is nevertheless not strictly frowned upon by the licensing authorities.

All the selected open drinking places were then categorized into three groups, urban, semi-urban and rural groups. This classification was based on local government funding allocation from the central government.

Sample size: We calculated the minimum sample size based on the sample size estimation for a descriptive cross-sectional study n = Z^2^pq/d^2^, where Z = 1.96, *p* = anticipated prevalence of harmful or hazardous drinking or possible dependence in open place drinking as (50%) in the absence of an earlier data on this population, q = 1-p = (50%), d = 0.05 (precision at 95% CI) [17]. This gave a sample size of 384.

However, we increased the minimum sample by 10% (34 participants) in anticipation of possible non-response. Thus, the minimum sample was 422. We approached 1507 out of a total of 1617 individuals counted for interview, 110 individuals were not approached because of obvious difficulty in following research protocol for reasons such as language barrier (69) and severe intoxication (41). However, 1393 gave consent, and all of them were interviewed as soon as operation of the open-drinking places began at about 6 pm (a response rate of 92.4%).

Although the minimum sample size required for the study was 422, interviewing 1393 automatically increased the power of the study.

During the study, we recruited all consenting subjects with non-probability purposive sampling and finalized data collection when all consenting subjects had been interviewed. The main characteristic of the purposive sampling was “open-drinking as the usual drinking location”.

In each of the study sites, the first participant was randomly selected and subsequent ones consecutively selected until all participants for the day were interviewed.

The number of participants selected at each study site is presented in Table 1. The minimum was 33 participants, the maximum was 89, mean (SD) number of participants across sites was 13.34 (6.64).

**Table 1:** Participants interviewed at each study site

Site number	n	%
1	41	3.7
2	40	3.6
3	36	3.2
4	38	3.4
5	44	3.9
6	38	3.4
7	43	3.8
8	42	3.8
9	39	3.5
10	45	4.0
11	33	2.9
12	37	3.3
13	36	3.2
14	33	2.9
15	38	3.4
16	37	3.3
17	84	7.5
18	76	6.8
19	81	7.2
20	81	7.2
21	88	7.9
22	89	8.0

### Data collection

The interviewers were senior registrars in psychiatry who had been involved in field surveys. At the beginning of the study, all interviewers received a 3-day training by the one of authors (VL), followed by another 2 days for debriefing and review after each interviewer had conducted two pilot interviews in the field. Three supervisors were appointed to make sure that the data collection was complete. Field checks were made to make sure the correct implementation of the protocol and full adherence to the interview format.

### Measures

#### Socio-demographic characteristics

Sociodemographic data were collected using a set of pre-tested and pre-coded questionnaire that yielded information on the socio-demographic characteristics such as age, sex, marital status, occupation, education, income, dwelling area. Dwelling area was classified into three groups, namely, urban, semi-urban and rural groups, based on local government funding allocation from the central government.

##### Prevalence of alcohol use and alcohol use disorders

The Alcohol Use Disorders Identification Test (AUDIT) was used to determine the prevalence of alcohol use and to identify persons with hazardous and harmful patterns of alcohol consumption. The AUDIT was developed by the World Health Organization (WHO) as a simple method of screening for excessive drinking and to assist in brief assessment [18, 19]. The AUDIT provides an accurate measure of risk across gender, age and culture [20]. The AUDIT has the advantage of having cross-cultural standardization, identifies harmful and hazardous alcohol use and is consistent with ICD 10 harmful alcohol use and dependence syndrome. It is very brief, rapid and flexible. Questions 1 to 3 captures hazardous alcohol use, 4 to 6, dependence and 7 to 10, harmful use.

A standard drink was defined as containing about 14 g of pure alcohol. This amount is found in about 350 mls of beer (12 oz) containing about 5% alcohol, about 145 mls (5 oz) of wine containing about 12% alcohol or about 45 mls (1.5 oz) of distilled spirits containing about 40% alcohol. We estimated 1 bottle of palm wine or sorghum as a standard drink, and ½ pint of local liquor or local brews as a standard drink.

Scoring: each question is scored from 0 to 4, the first response for each question, for example, is “never”, scoring 0, the second (less than monthly) scoring 1, the third (monthly) scoring 2, the fourth (weekly) scoring 3, and the last response (daily or almost daily) scoring 4. In the case of questions 9 and 10, which only have three responses, the scoring is 0, 2 and 4 (from left to right). A total score of 8 or greater is associated with harmful or hazardous drinking in men and women, a score of 13 or more in women, and 15 or more in men, indicates likely alcohol dependence. Among respondents older than 65 years, a cut - off point of 7 is recommended.

In this study, scores of 8 and above which signified harmful or hazardous alcohol use and possible dependence were defined as “likely” alcohol use disorder (AUD).

Range of AUDIT Scores and Specific Intervention: scores of 0 to 7 were categorized as zone I in terms of health risk. Scores between 8 and 15 were categorized as zone II in terms of health risk, scores between 16 and 19 were categorized under zone III health risk while a score of 20 and above was categorized under zone IV risk level.

##### Prevalence of smoking

Data on current smoking was obtained from the question: “in the past 4 weeks, how often have you smoked cigarettes?” Responses were “never”, “once or twice”, “weekly”, and “daily/almost daily”. For the purpose of this study, any response, other than never, was considered to be current smoking.

##### Pre-test

A pre-test was carried on 150 participants (not part of the study sample) that showed that all instruments of data collection had a good acceptability by the participants selected for the pre-test and a short administration time. We assessed “acceptability” using six component constructs: attitude towards the contents of the instruments, a dropout from the study, willingness to participate, satisfaction with participation in the study, perceived consequences of being part of the research and adherence to research protocols [21], of which the average score of the participants was over 90% in each of these constructs.

### Analysis

The associations between likely AUD and demographic characteristics were sought using Chi square statistics. Binary regression analysis was used to determine the effects of multiple confounding variables with the outcome variable, likely AUD. The variables selected for regression analysis were those that were significantly associated with likely AUD during the univariate analysis, these were: age, religion, employment and smoking. We adjusted for both age and gender because of their known associations with alcohol consumption. All tests of significance were set at 95% confidence interval, *p* <  0.05. Data analyses were by the Statistical Package for the Social Sciences (SPSS) version 20.0 software. The datasets used and analyzed during the current study are available from the corresponding author on reasonable request.

## Results

Out of all the 1393 subjects interviewed, data were complete for 1119. Therefore, analysis was performed among 1119 respondents. The mean age (SD) of all respondents was 39.10 (12.06) years (Not in any Table). The sociodemographic characteristics of the respondents are presented in Table 2.

**Table 2:** Sociodemographic characteristics of drinkers

Age group	N	%
< 25	113	10.1
25–34	325	29.0
35–44	316	28.2
45–54	231	20.6
55–64	104	9.3
> 64	30	2.7
Gender		
Male	826	73.8
Female	293	26.2
Education		
No Formal	78	6.9
Elementary	286	25.6
Secondary	253	22.6
Post- secondary	502	44.9
Employment		
In Employment	957	85.5
Unemployed	162	14.5
Dwelling Area		
Urban	308	27.5
Semi-urban	312	27.9
Rural	499	44.6
Marital Status		
Married	634	56.7
Not Married	484	43.3
Religion		
Islam	109	9.7
Christianity	1010	90.3
Ethnicity		
Yoruba	658	58.8
Igbo	235	21.0
Hausa	36	3.2
Others	190	17.0
Current Smoking		
Yes	714	63.8
No	405	36.2

Out of the 1119 respondents, 124 (11.1%) were abstainers; the prevalence of current drinkers was 995 (88.9%), while the prevalence of a likely AUD (zones II-IV) was 39.5% (Table 3).

**Table 3:** Prevalence of drinking and risk level (1119 Respondents)

Risk Level	AUDIT Score	Alcohol Use	n	%
Zone 1	0	Abstainers	124	11.1
Zone 1	1–7	Low risk users	553	49.4
Zone II	8–15*	Likely AUD	297	26.5
Zone III	16–19*	Likely AUD	76	6.8
Zone IV	≥20*	Likely AUD	69	6.2

*: Score > 7 is indicative of Likely Alcohol Use Disorder (AUD)

Out of the 995 current drinkers, the prevalence of a likely AUD (zones II-IV) was 44.4% (Table 4).

**Table 4:** Risk level among current drinkers (995 Respondents)

Risk Level	AUDIT Score	Alcohol Use	n	%
Zone 1	1–7	Low risk users	553	55.6
Zone II	8–15*	Likely AUD	297	29.8
Zone III	16–19*	Likely AUD	76	7.6
Zone IV	≥20*	Likely AUD	69	6.9

*: Score > 7 is indicative of Likely Alcohol Use Disorder (AUD)

Out of the current drinkers, the mean age (SD) of respondents with a likely AUD was 40.77 (12.67) and that of those respondents without a likely AUD was 38.62 (11.60). The mean age of respondents with a likely AUD was significantly higher than the mean age of those without AUD, *t* = 2.8, df (993), *p* = 0.005 (Not in any table).

Univariate analysis showed that the prevalence of a likely AUD increased with increasing age, X^2^ = 20.9, *p* = 0.001.

Post-hoc multiple pairwise comparisons showed that the significant age difference in the prevalence of a likely AUD was due to a lower prevalence of a likely AUD among respondents < 25 years of age compared with respondents 35–44 years of age, compared with respondents 45–54 years of age, and when compared with respondents 55–64 years of age, X^2^ = 11.9, *p* <  0.001, X^2^ = 11.7, *p* <  0.001, X^2^ = 11.6, *p* <  0.001 respectively on one hand, and the prevalence of a likely AUD also being lower among respondents between 25 and 34 years when compared with those respondents 35–44 years, compared with those respondents 45–55 years, and when compared with those respondents 55–64 years, X^2^ = 4.3, *p* = 0.03, X^2^ = 4.4, *p* <  0.04, X^2^ = 4.8, *p* = 0.02 respectively, on the other hand. There were no significant age differences in the prevalence of a likely AUD after the age of 35 years (Not in any Table).

The prevalence of likely was also higher in men, X^2^ = 38.7; *p* <  0.001; among the Christians, X^2^ = 49.3, *p* <  0.001; among the employed, X^2^ = 9.8, *p* = 0.002; among smoker, X^2^ = 11.6, *p* = 0.001 and higher in the rural setting, X^2^ = 13.5, *p* = 0.001 (Table 5). Post-hoc pairwise comparisons show that there was a significantly higher prevalence of likely AUD in the rural areas compared with the urban areas X^2^ = 8.6, *p* = 0.003 and also between rural areas and semi-urban areas X^2^ = 8.9, *p* = 0.003 (Not in any Table).

**Table 5:** Univariate and multivariate characteristics of likely Alcohol Use Disorder (AUD) (Prediction 64.1%)

	Univariate Analysis						Multivariate Analysis			
Demography/	Likely AUD				X^2^	P	Likely AUD			
Clinical Variable	Yes		No					95% CI		
	n	%	N	%			OR	Lower	Upper	P
Age										
< 25	32	25.2	95	74.8	20.9	0.001^BS^		Adjusted		
25–34	108	34.8	202	65.2						
35–44	142	43.3	186	56.7						
45–54	99	44.2	125	55.8						
55–64	47	48.0	51	52.0						
> 64	14	43.8	18	56.3						
Gender										
Male	371	44.9	455	55.1	38.7	< 0.001		Adjusted		
Female	71	24.2	222	75.8						
Religion										
Christianity	433	42.9	577	57.1	49.3	< 0.001	1			
Islam	9	8.3	100	91.7			0.13	0.06	0.26	< 0.001
Ethnicity										
Igbo	111	43.5	144	56.5	7.3	0.1		NA*		
Yoruba	210	37.8	346	62.2						
Middle Belt	97	37.7	160	62.3						
Hausa	11	36.7	19	63.3						
Minorities	13	61.9	8	38.1						
Years of Education										
None	114	45.1	139	54.9	5.0	0.2		NA*		
1–6	196	39.0	306	61.0						
7–12	105	36.7	181	63.3						
> 12	27	34.6	51	65.4						
Marital Status										
Married	259	40.9	375	59.1	1.2	0.3		NA*		
Unmarried	182	37.6	302	62.4						
Employment										
In Employment	396	41.4	561	58.6	9.8	0.002	1			
Unemployed	46	28.4	116	71.6			1.30	0.86	1.96	0.4
Residence										
Urban	107	34.7	201	65.3	13.5	0.001^BS^	1			
Semi-urban	108	34.6	204	65.4			0.96	0.68	1.40	0.8
Rural	227	45.5	272	64.5			1.84	1.34	2.53	< 0.001
Current Smoking										
No	133	32.9	271	67.1	11.6	0.001	1			< 0.001
Yes	309	43.3	405	56.7			1.81	1.37	2.40	

*NA: Not included in the regression equation because of lack of significant associations between these variables and Likely Alcohol Use Disorder (AUD) during univariate analysis; *BS*: Bonferonni Significant

Multivariate analysis showed that Islamic religion was a negative predictor for a likely AUD, OR = 0.13, 95% CI (0.06–0.26), while rural residence, OR = 1.84, 95% CI (1.34–2.53) and cigarette smoking OR = 1.81, 95% CI (1.37–2.40) were positive predictors. These factors accounted for 63.1% of the variations due to AUD (Table 5).

## Discussion

The survey research reported herein has investigated the relationships between alcohol patrons and open-places. Thus, our findings were discussed within these contexts. Conceivably, it is often difficult to obtain adequate samples of people who drink within specific contexts from general population surveys. Therefore, location-based sampling, in which participants are recruited from those contexts, offers an attractive alternative [22].

### Prevalence of AUD

Specifically, almost four in every ten respondents (39.5%) had likely AUD. This figure is about 13 folds higher than the WHO report for Africa [23]. When considered in the context of the drinking population, the prevalence of a likely AUD was 44%. This figure is close to that reported (almost 50%) that similarly used AUDIT, in a high-prevalence alcohol use setting (nightclubs) in Brazil in 2015 [24]. However, compared with figures from the general population, the prevalence of likely AUD reported in the study being reported was high. For example a figure of 29.2% was reported in a community survey in India [25] and 18.2% was reported in Brazil [26]. A much lower fig. (2.8%) was reported among the Bhutanese refugees in Nepal [27]. The relatively high prevalence of a likely AUD in this study could be adduced to several reasons. First, the open-space drinking context may attract people with similar specific characteristics to creating unique niches for drinking.

Social ecological theories of alcohol use focus upon the specific roles that drinking contexts play in the aetiology of alcohol use and related problems. For example, the “niche theory” emphasizes that some drinkers create a unique drinking niche, such as less supervised places, including parking lots or street corners, while other drinkers with lower levels of income may be less likely to drink at restaurants or bars where alcohol is more expensive. On the other hand, the “assortative drinking” drinkers take their alcohol in contexts in which they find people like themselves [28]. Thus, these drinkers may self-select into contexts that provide access to alcohol in an environment best enjoyed by them.

In the current study, 995 out of 1119 (88.9%) recruited individuals were current drinkers. This is a high drinking population and could be the potential explanation of the high rate of a likely AUD in our sample. Nevertheless, considered in the light of AUDIT being a screening instrument and not diagnostic, the prevalence of authentic AUD may be lower than the figures reported in the current study.

Specifically, out of sample, almost a half (49.4%) scored between 1 and 7 on the AUDIT, while one in every nine (11.1%) were abstainers (AUDIT score 0), these individuals were in Zone I (low health risk). This finding has implications for timely intervention to prevent them from developing a dependence syndrome over time. In a community study in the south-south zone of Nigeria, an area branded as having the highest prevalence of alcohol consumption, 63.6% of respondents were found to be in zone I using the AUDIT [8].

We also found that almost 26.5% of the respondents were in Zone II, indicating harmful or hazardous alcohol use. Brisibe and colleagues found that 36% of their samples were in zone II of the AUDIT [8]. Thus, the proportion of drinkers with harmful or hazardous drinking in the current study is lower than the drinking rate reported among the high alcohol consumption population of south-south Nigeria [8]. Although about 7% of the respondents were in Zone III indicating high-risk drinkers, over 6% were in Zone IV, indicating a possible dependence. A much higher fig. (14.2%) was reported by Briside and colleagues in south-south Nigeria for respondents in Zone IV [8].

Usually, prevalence of alcohol use disorders varies widely because of the definition and the instrument used in the definition, the age group which was studied, setting of the study and the methodology which was adopted for the sampling. We argue that our sample may be at a high health risk that would require intervention.

### Demographic associates of AUD

The sociodemographic characteristics of the respondents were quite remarkable. The majority of the respondents were young adults and those in their middle ages. This is in support of previous studies [10]. Nevertheless, the lowest proportion of respondents with a likely AUD was in the younger age group, below 35 years. Therefore, the sample with likely AUD comprised of men and women within the productive age, rather than adolescents, who generally favour drinking in concealed environments such as bars, pops and residential halls [29]. This may account for the lower proportion of respondents with a probable AUD being of the younger age group.

We found that AUD was more prevalent in the older age groups. Although contrary to research reports indicating that some individuals show dramatic reduction in problem drinking with age [30], our finding is in support of a recent research evidence showing that older people are more likely to have consumed alcohol in the last week than those who are young [31]. The observed association between AUD and older age has a number of potential reasons, firstly, older adults are more likely to develop AUD as a result of life changes such as retirement [32] or bereavement, or feelings of boredom, loneliness [33], loss of job and depression [34].

We also found that AUD was almost confined to men. Given the preponderance of men in the drinking population, this could be an artifact, Research reports have consistently shown that men not only drink more than women [2, 3, 10, 35], they are also more likely to have alcohol-related harm [36]. This may in part reflect the observation that women tend to become easily intoxicated compared to men, given the same equivalent dose of alcohol because despite being faster alcohol metabolizers [37]. However, we should be mindful that gender differences in problem drinking are modified by cultural and not just biological factors [38]. Given that self-restraint of drinking by women in some culture signifies their roles as social guardians [39], women are less likely to be engaged in public drinking than men.

While, there are reports that problem drinkers were more likely to be unmarried [40], some other literatures have argued that problems drinkers were more common among married men and only in unmarried women [41]. However, we did not find such an association. This finding may suggest that open-door drinkers have peculiar characteristics, perhaps different from other population of drinkers.

Consistent with reports from different parts of the world [3, 10, 42], the vast majority of those with likely AUDs were Christians. Although alcohol is generally abhorred by people with deep religious affiliation irrespective of the religion being practiced, the Islamic religion tends to exhibit more clarity on alcohol use, which is abstinence [42].

Although the general notion is that problem drinking is more prevalent in unemployed people [43, 44], our result was contrary to this. The question is what exactly could be responsible for this paradox? Our finding may suggest that employment status was a function of economic strength to purchase alcohol. Indeed, there is a direct relationship between economic viability and higher levels of drinking [45, 46].

We also found that rural dwellers were significantly more likely to have AUD. This finding fits into previous research findings that drinking is more prevalent in rural areas [2, 3, 47]. It is likely that being a rural dweller could be associated with less likelihood of obtaining adequate health information about the health implications of excessive drinking.

Consistent with abundant research findings was our observed association between smoking and a likely AUD. The co-use of alcohol and tobacco is very common irrespective of the study type [48, 49]. Open places are often one of the common places where people, especially young adults, congregate to drink and smoke. The strong, rewarding effects of nicotine paired with alcohol [50], is a major factor responsible for co-use of tobacco and alcohol, even for occasional and light smokers. The discussion on co-use of alcohol and tobacco, however, is outside the scope of the current study.

#### Abstainers

About 1 in every 10 respondents (11.1%) was an abstainer. While it is notable that a handsome number of people who patronize open drinking places were not drinkers, we conceive that this group of individuals is peculiar because they belong to the zone I of health risks who would require brief intervention. This is so because of their close relationship with drinkers. Studies have demonstrated that peer use of alcohol was found to be predictive of problematic alcohol use [51, 52]. Taken together, peer alcohol use is an important factor in the transition from abstainers to users. To corroborate this, there are reports that social network and peer effects are important determinants of drinking [53].

#### Policy implications

Our findings are expected to translate into policy where the purchase of alcohol is tied to on-the-spot breath alcohol content, and the higher the breath content, the more a consumer pays, up to a certain level where he may not be able to buy more [54]. This may also lead to legislations mandating beer manufacturer to display educational materials on the harmful effects of unsafe alcohol consumption at public drinking places.

Laws need to be put in place and enforced regarding advertisement, manufacture or sale of alcohol in open places, labeling of the alcoholic concentration in local brews with the approval of the appropriate authority such as the National Agency for Food and Drug Administration and Control. The implementation of effective policies that will reduce harmful and hazardous alcohol consumption requires a good understanding of the policy development process and which strategies are likely to work with good public support. For example, certain WHO specific policy areas: regulating alcohol marketing; restricting alcohol sales, regulation of retail sales of alcohol; alcohol taxation and controls on alcohol packaging; strengthening drinking and driving laws; strengthening health sector response; and raising political commitment [55]. Unfortunately, these policies are poorly implemented in Nigeria.

The current study has a number of limitations. There are usually methodological and reporting shortcomings using location-based sampling. This purposive sampling is highly vulnerable to errors in judgment by the researcher. The reliability of the results may be low, and the selection process is subject to a lot of bias. Considered in light of these, findings from studies using location-based sampling may not be generalizable to the universe of patrons or open drinking places across other parts of the country. Notwithstanding this, the potential strengths of location-based sampling over other possible strategies remain important. It is cost-effective and time-effective. The sampling method is also the only feasible method with a limited number of primary data source. It also has the advantage of exploring this contextual drinking from an anthropological angle.

Also, obtaining information from individuals who could have been intoxicated is highly subject to significant errors. Furthermore, the AUDIT is a screening instrument, thus the prevalence of AUD reported in the current study is likely to be an overestimation.

## Conclusion

In conclusion, a significantly high proportion of social drinkers in open places are problem drinkers who require intervention. Attention should be given to this population as they constitute the economically viable and productive segment of the society, yet they have little alcohol and substance-related health education. Our study has policy implication regarding outdoor-open space drinking that is the common drinking trend in Nigeria. Patrons require health-educational awareness programs on the harmful effects of excessive drinking.

## Abbreviations

AUD: Alcohol use disorder
AUDIT: Alcohol Use Disorder Identification Test
LGA: Local Government Area
PI: Principal Investigator
